# Livestock-associated *Staphylococcus aureus* in Childcare Worker

**DOI:** 10.3201/eid1704.101852

**Published:** 2011-04

**Authors:** Erin D. Moritz, Tara C. Smith

**Affiliations:** Author affiliation: University of Iowa, Iowa City, Iowa, USA

**Keywords:** Staphylococcus aureus, livestock, MRSA, carriage, zoonosis, child care, daycare, occupational health, bacteria, letter

**To the Editor:** Carriage of *Staphylococcus aureus* sequence type (ST) 398 has primarily been reported as occurring among persons in contact with livestock, including swine and cattle (*1*,*2*). This association has given rise to the characterization of this strain as livestock associated (*3*). However, ST398 colonization or infection in persons lacking identified livestock-associated risk factors have been reported (*4*,*5*). We report ST398 colonization in a childcare worker in Iowa, USA.

As part of a surveillance study of *S. aureus* carriage in child daycare facilities, samples were collected from employees, children, and environmental surfaces. Nasal samples were taken from participating children, and nasal and pharyngeal samples were taken from participating employees. All samples were cultured, and *S. aureus* isolates were examined by pulsed-field gel electrophoresis, *spa* typing, and antimicrobial drug susceptibility testing and tested for the Panton-Valentine leukocidin gene. One participant was colonized in the nose and throat with *t*571, a *spa* type previously reported to correspond to ST398 (*1*). The isolates were nontypeable when *Sma*I was used, also a characteristic of ST398 (*6*). They were digested with *Cfr*9I and found to be closely related to an ST398 isolate of *spa* type *t*034 of swine origin but distinct from *S. aureus* isolated from 2 other employees at the facility (Figure). Both ST398 isolates were susceptible to methicillin.

**Figure:**
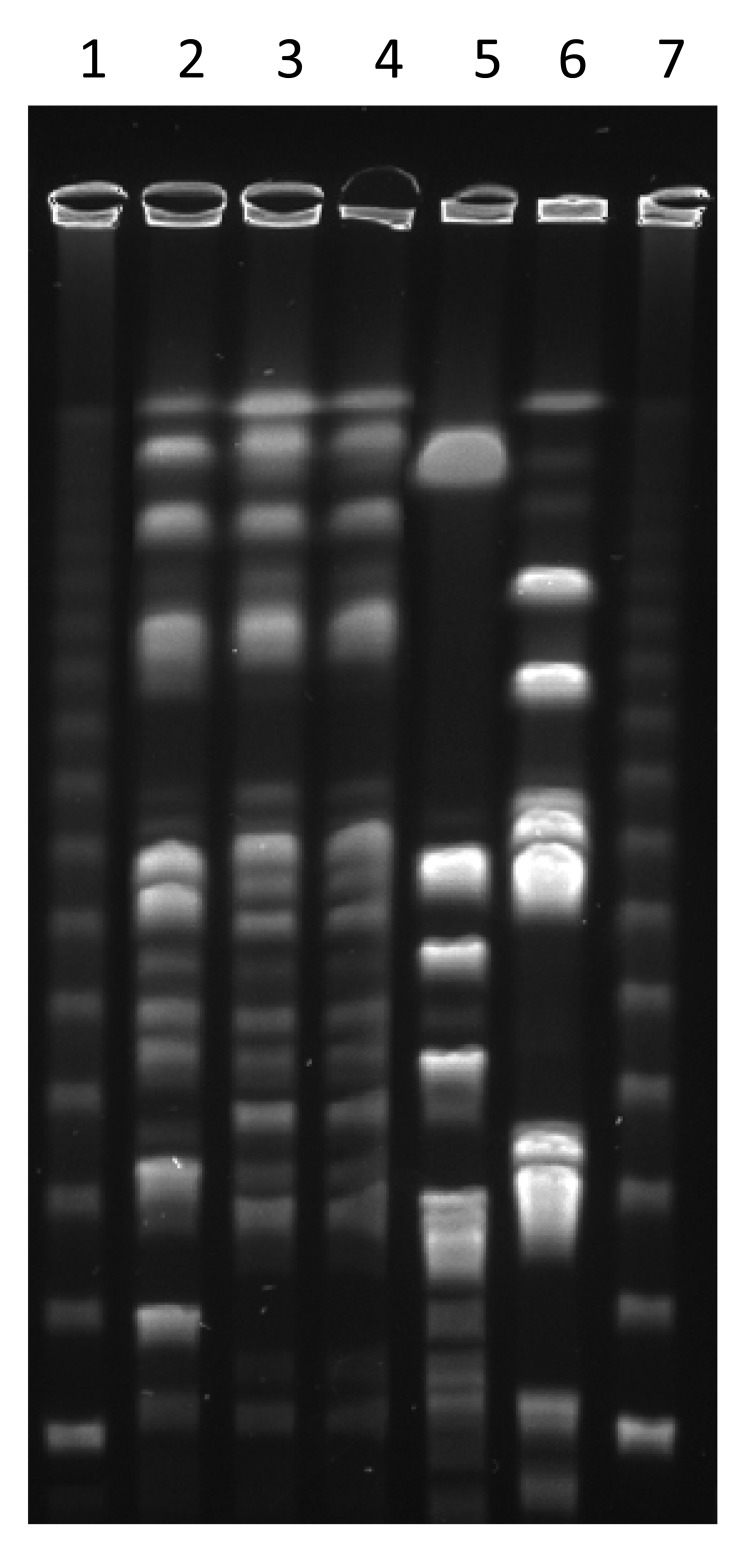
Pulsed-field gel electrophoresis of *Staphylococcus aureus*. Isolates were digested with *Cfr*9I. Lanes 1 and 7, molecular mass ladder; lane 2, *t*034 sequence type (ST) 398 isolate from pig; lane 3, *t*571 ST398 nasal isolate from colonized childcare employee; lane 4, *t*571 ST398 throat isolate from colonized childcare employee; lanes 5 and 6, non-ST398 isolates (*t*2228 and *t*084, respectively) from 2 other childcare employees.

The colonized employee was a 24-year-old woman who had worked at the facility for ≈5 years. She reported a history of melanoma but was not currently taking any chemotherapy drugs and had not been hospitalized in the previous 12 months. She reported having a family member who worked in a hospital and had direct contact with patients, but the employee lived alone and responded negatively to questions about whether she or immediate family members had had contact with animals or worked in a processing plant.

ST398 may be transmitted from livestock to community members and then from person to person. It can potentially be transmitted in food; several studies have documented ST398 in raw meats (*7*,*8*), and we identified this strain in retail meat products in Iowa (T.C. Smith et al., unpub. data). Secondary transmission of ST398 from colonized persons to contacts has also been suggested, but the few publications reporting this suggest that ST398 seems to be less transmissible by this route than are common human strains (*9*).

We cannot be sure whether either of these routes played a role in acquisition of ST398 by this employee. Although no other tested persons in this childcare facility were found to carry ST398, only 24 (40%) of the 60 employees and 8 (4.8%) of the 168 children participated, suggesting the possibility of a reservoir in the facility among those who were not tested. Of the 24 employees who participated, 2 reported occupational contact with any animals, 2 reported contact with swine, and 3 reported contact with cattle. However, no participant reported having animals other than cats or dogs on their property. It is possible that >1 sampled employee may have been a transient ST398 carrier but negative at the time of our sampling.

Reports of ST398 in persons who had no direct contact with livestock in the United States are rare (*10*). To provide a better understanding of the epidemiology of this novel strain, further examination of the emergence of this isolate in community settings and on farms is needed.
